# Sarcoidosis: A Mimicker of Ocular Cicatricial Pemphigoid

**DOI:** 10.7759/cureus.93862

**Published:** 2025-10-05

**Authors:** Ricardo A Murati Calderon, José J López-Fontanet, Estefania Ramirez Marquez, Ana S Tavares Reigada, Armando Oliver

**Affiliations:** 1 Department of Ophthalmology, School of Medicine, Medical Sciences Campus, University of Puerto Rico, San Juan, PRI; 2 Pathology, Institute of Immunofluorescence, Dermpath Diagnostics, Pompano Beach, USA

**Keywords:** ocular cicatricial pemphigoid, ocular cicatrizing conjunctivitis, ocular sarcoidosis, pseudopemphigoid, sarcoidosis

## Abstract

We report a rare case of ocular sarcoidosis presenting as bilateral cicatricial conjunctivitis, initially misdiagnosed as ocular cicatricial pemphigoid (OCP). A 69-year-old woman presented with a five-year history of chronic bilateral ocular irritation, trichiasis, conjunctival injection, and progressive cicatrization. She had previously undergone a conjunctival biopsy at an outside facility several years prior, which was reported without abnormal findings. Despite treatment with topical cyclosporine, artificial tears, and dapsone for presumed OCP, her symptoms persisted. A repeat conjunctival biopsy was eventually performed and revealed nodular epithelioid granulomatous inflammation with stromal fibrosis and scattered Langhans-type giant cells, consistent with ocular sarcoidosis. There was no prior history or systemic evidence of sarcoidosis. The patient was started on oral prednisone with a structured taper and mycophenolate mofetil. At the six-month follow-up, she reported marked improvement in ocular discomfort, with stabilization of cicatricial changes. This case highlights the importance of considering sarcoidosis in the differential diagnosis of chronic cicatrizing conjunctivitis, even in the absence of systemic findings, and underscores the diagnostic role of conjunctival biopsy in guiding management.

## Introduction

Sarcoidosis is a systemic inflammatory disease marked by the formation of non-caseating granulomas in affected tissues [1]. This condition mainly affects people from 20 to 40 years old, showing a slight female predominance and a higher occurrence rate in African American populations [2]. The clinical diagnosis of sarcoidosis is generally supported by the involvement of characteristic organ sites, distinctive radiologic patterns, and histological confirmation through biopsy [3].

Ophthalmic manifestations of sarcoidosis can affect any part of the eye and its adnexa [4]. These manifestations often include conjunctival granulomas, optic neuritis, and lacrimal gland involvement [4]. Among these, uveitis stands out as the most common ocular manifestation, occurring in up to 70% of cases [5]. This is particularly noteworthy as ocular involvement is the presenting symptom in 30-40% of patients diagnosed with systemic sarcoidosis [5,6].

Cicatricial conjunctivitis has been more commonly associated with ocular cicatricial pemphigoid (OCP), a form of mucous membrane pemphigoid characterized by chronic, relapsing-remitting bilateral conjunctivitis [7,8]. However, few cases of cicatricial conjunctivitis are reported in patients with sarcoidosis [9]. Treatment options include topical or systemic steroids, nonsteroidal anti-inflammatory agents, topical cyclosporine, antimalarials, and disease-modifying antirheumatic drugs [10,11]. The choice of treatment depends on the severity of the disease and specific ocular manifestations. We herein present a 69-year-old woman with chronic conjunctival inflammation initially suspected to be OCP but ultimately diagnosed as ocular sarcoidosis following biopsy and further investigation. This case highlights the importance of considering sarcoidosis in the differential diagnosis of chronic ocular inflammatory conditions that mimic OCP.

## Case presentation

A 69-year-old woman was referred to the clinic with a five-year history of bilateral ocular irritation, swelling, itchiness, and intermittent secretions. She denied any associated pain, diplopia, loss of vision, or any other ocular complaint. The patient had a past medical history, including asthma and diabetes mellitus type 2. The ocular history was remarkable for chronic dry eye syndrome, chronic conjunctivitis in both eyes (OU), and a normal conjunctival biopsy five years prior, as recalled by the patient. Current ocular medications included cyclosporine ophthalmic emulsion 0.05% twice daily in both eyes and artificial tears every two hours in both eyes. Allergies included acetaminophen and oxycodone. Review of systems and family history was unremarkable. Social history was notable for the prevalence of smoking cigarettes.

**Figure 1:**
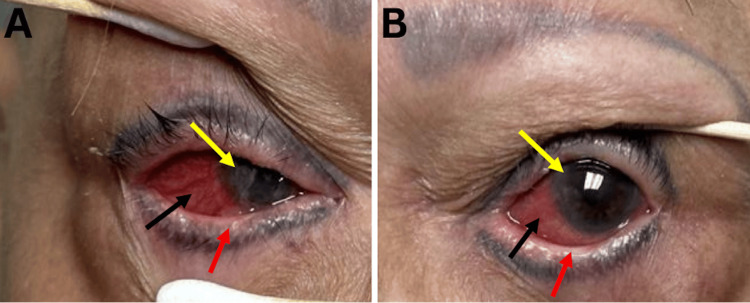
External photographs of both eyes External photographs of the right eye (A) and left eye (B) demonstrate chronic conjunctival inflammation with diffuse bulbar conjunctival injection (black arrows), subepithelial fibrosis, and marked conjunctival scarring extending to the cornea (yellow arrows). Additional findings more prominent on slit-lamp examination include lower fornix foreshortening (red arrows), symblepharon formation, and extensive corneal conjunctivalization, consistent with advanced cicatricial conjunctivitis.

Upon comprehensive ophthalmic evaluation, her best-corrected visual acuity was counting fingers in the right eye (OD) and 20/400 in the left eye (OS). The pupils were round and reactive to light, and there was no afferent pupillary defect in either eye. The intraocular pressure was 17 OD and 19 OS. Ishihara color plates could not be assessed due to the patient’s limited vision. Extraocular movements were within normal limits in both eyes. Slit lamp examination revealed trichiasis OU, shortening of the fornix OU, nasal and temporal symblepharon around OU, bilateral diffuse injection 2+, a central epithelial defect in OU, and corneal conjunctivalization OU (Figure 1A, 1B). The patient's fundus examination was limited; however, it was grossly unremarkable.

**Figure 2:**
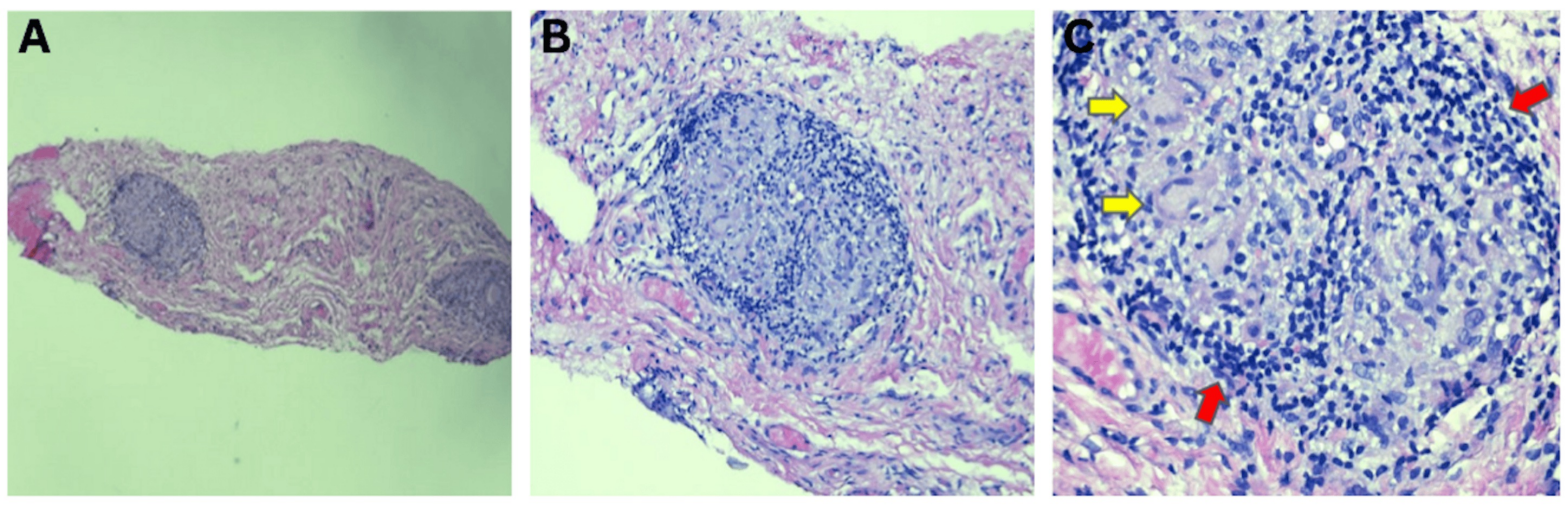
Histopathological slides Conjunctival biopsies across all slides reveal submucosal, discrete, nodular granulomas. (A) Submucosal discrete nodular histiolymphocytic aggregates (H&E 5x). (B) Submucosal nodular epithelioid granuloma crowned by relatively few lymphocytes (H&E 20x). (C) Submucosal discrete nodular epithelioid granuloma containing Langhans-type multinucleated histiocytes (yellow arrow), with few lymphocytic crowning (red arrow). Caseous necrosis, polymorphonuclear cells, and foreign bodies are not identified (H&E 20x). H&E: hematoxylin-eosin stain

Lab studies, including complete cell blood count, comprehensive metabolic panel, urine analysis, QuantiFERON Tb gold, hepatitis panel, angiotensin-converting enzyme, and lysozyme, were all within normal limits. Chest X-ray revealed thoracic spondylosis, but no other findings such as hilar lymphadenopathy or parenchymal infiltrates were found. The patient was started on serum tears 20% and dapsone 100mg daily due to clinical concern for OCP. Conjunctival biopsy was delayed due to the patient’s social circumstances. However, once performed, the biopsy revealed nodular epithelioid granulomatous inflammation with stromal fibrosis and mild squamous metaplasia (Figure 2). The granulomas were composed of histiocytes, including foamy cells and scattered Langhans-type multinucleated giant cells, with crowning lymphocytes. No necrobiosis, vasculitis, or central necrosis was identified. Special stains (PAS, AFB) and polarization studies were negative for fungi, acid-fast bacilli, and refractile material. The differential considerations included OCP, infectious granulomatous conjunctivitis, foreign-body reaction, granulomatosis with polyangiitis, and granulomatous rosacea or lipogranuloma. These were excluded based on negative immunofluorescence and stains, the absence of necrosis or exogenous material, and the lack of characteristic histologic features, including the absence of prominent lipid vacuoles or neutrophilic suppuration. The finding of well-formed, non-caseating epithelioid granulomas with scattered Langhans-type giant cells and negative infectious stains supported a diagnosis of sarcoidosis.

Consistent with the diagnostic principles of the American Thoracic Society and the World Association for Sarcoidosis and Other Granulomatous Disorders, the compatible presentation, non-caseating granulomas on tissue, and exclusion of alternative causes establish these findings as biopsy-proven ocular sarcoidosis involving the conjunctiva. Using the WASOG Organ Assessment Instrument, ocular involvement is classified as highly probable. The diagnosis was therefore revised from OCP to ocular sarcoidosis. Subsequently, the patient discontinued dapsone and was started on mycophenolate mofetil (CellCept) 1.5 g orally twice daily and prednisone 60 mg orally once daily, followed by a weekly tapering schedule.

At the six-month follow-up, the patient reported marked improvement in ocular discomfort, including burning sensation and itchiness OU, compared to the initial presentation. Following the revised diagnosis of ocular sarcoidosis and initiation of treatment with mycophenolate mofetil and oral prednisone, the patient demonstrated significant clinical improvement. On ophthalmic examination, there was a notable reduction in conjunctival and scleral injection, along with stabilization and partial regression of cicatricial changes. The patient has remained clinically stable on the current immunosuppressive regimen. 

## Discussion

Sarcoidosis is widely recognized as a cause of uveitis; however, it has rarely been described in association with cicatricial conjunctivitis [12-14]. Since cicatricial conjunctivitis may occur in patients without a prior history or concurrent symptoms of systemic sarcoidosis, a broad differential diagnosis must be explored. The most common etiology of cicatricial conjunctivitis is mucous membrane pemphigoid [8,15]. Other pathologies that may mimic the ocular manifestations of mucous membrane pemphigoid include ocular rosacea, chemical burns, toxicity of topical medications, lichen planus, atopy, trauma, and autoimmune diseases such as Stevens-Johnson's syndrome [16,17]. The precise etiology of cicatricial conjunctivitis may be obtained by performing a conjunctival biopsy. Sarcoidosis may be histopathologically distinguished by the presence of non-caseating granulomas [18,19].

The patient described in this case did not have any prior signs or symptoms related to sarcoidosis. She had an unremarkable workup including a complete cell blood count, comprehensive metabolic panel, urine analysis, QuantiFERON Tb gold, hepatitis panel, angiotensin-converting enzyme, lysozyme, and a chest X-ray. According to the patient, she had previously undergone a conjunctival biopsy, which was unremarkable. It is possible that the diagnosis of sarcoidosis was overlooked, given its rarity in such instances. Nevertheless, another biopsy was performed, which confirmed the diagnosis of sarcoidosis.

The appropriate management of a patient with cicatricial conjunctivitis secondary to sarcoidosis involves further workup to determine if there are any systemic manifestations. Treatment options include topical or systemic steroids, nonsteroidal anti-inflammatory agents, topical cyclosporine, antimalarials, and disease-modifying antirheumatic drugs [11,20]. The patient described in this case had been treated with preservative-free artificial tears, cyclosporine ophthalmic emulsion 0.05%, serum tears 20% and dapsone 100 mg daily. Despite this regimen, clinical progression of the disease was noted. A repeat conjunctival biopsy was performed, which revealed well-formed non-caseating granulomas consistent with ocular sarcoidosis. Based on these findings, the patient was started on mycophenolate mofetil 1.5 g twice daily and oral prednisone 60 mg daily. A structured weekly tapering regimen was implemented and closely monitored during follow-up appointments, leading to stabilization of the cicatricial process. Hence, as with other ocular inflammatory diseases, therapeutic decisions must be continually reassessed in light of evolving clinical and histopathologic data. 

## Conclusions

In rare instances, cicatricial conjunctivitis may be secondary to sarcoidosis. This case suggests the cicatricial conjunctivitis secondary to sarcoidosis may occur in the absence of systemic signs or symptoms of sarcoidosis. Strategies for managing this condition involve a personalized approach that considers exploring each patient’s individual therapeutic threshold. 
